# More about Public Laundries

**Published:** 1884-07

**Authors:** 


### EDITORIAL.

---

MORE ABOUT PUBLIC LAUNDRIES.—There is a melancholy species of poor satisfaction to be derived from the contemplation of the woes of a neighbor whose sufferings have been profoundly experienced in our own person. This frank admission of the lower instincts of a depraved human nature, is suggested by glancing over the published report of the late General Medical Council of Great Britain. In one point, and in one point only, does this report suggest the transactions of some of our State Boards of Health. The sole point of resemblance is the opportunity, which is here improved, to do the washing of some soiled linen in public. If it is painful for us who live in the State of Illinois to have the mass of British practitioners understand that the professional men of the Northwest do not know how to spell, it may be scarcely less disagreeable to English gentlemen to have the inhabitants of the settlements in the wild west impressed with the conviction that the average British practitioner is a person capable of setting fire to property, of personating other individuals, or of being guilty of other crimes for which the penalty of the law has been inflicted behind the bars of a government penitentiary.

For it is a melancholy fact that in the report of the Council to which we refer, one Sadgrove had his name removed from the Register "on account of having pretended to have qualifications which he did not possess." (How many names, alas, would disappear from the roster of great men in science, if this rule were successfully and rigidly enforced in all cases!)

At the same meeting, one Story was refused restoration to the Register because, in the year 1881, he had been convicted of arson for which he had suffered two years imprisonment. Again, one Smith (and even this not unfamiliar name persuades us that in many respects, indeed, the practitioners on the other side of the Atlantic are truly our brethren after the flesh, with a tendency for the same imposthumes to develop upon their otherwise vigorous bodies!) who had lost registration in 1873 on account of "infamous conduct," was restored by the Council at this same meeting on the petition of a large number of medical practitioners. A curious case was presented in the person of one O'Hare, who had personated a registered practitioner named O'Hara. O'Hare, it seems, had suffered erasure from the Register in 1878, on account of "felony;" while the man whose name he had assumed had also been dropped from the Register in consequence of failure to reply to certain letters which had been addressed to him. One Murray also suffered erasure from the Dentists' Register on account of "infamous conduct."

From certain recommendations, made by the committee also the idea is conveyed that at the examination of candidates for license to practice, some who were capable of successfully passing the examination had been guilty of "personating" those who were not capable of undergoing the ordeal, thus securing the license for the latter. We sincerely hope that the announcement of this fact in this public manner will not stimulate Yankee ingenuity to the perpetration of a still more refined method of outwitting law.

We beg leave to assure our British brethren, with sincerity and a due degree of emphasis, that while on this side of the

Atlantic we do not all know how to spell, there is, in the language of their countryman, Mr. Matthew Arnold, "a remnant" of us who do. Though we understand from the report of their General Medical Council that there are British practitioners in the United Kingdom who have been guilty of "arson," "infamous conduct," "felony," "personation," and divers other eccentricities, some of which have been punished by imprisonment, we are firmly convinced, and we say it without fear of contradiction, that there is "a remnant" of British practitioners who have not been guilty in these particulars.

Critics more invidious and less magnanimous than they should be, might be tempted here to make some odious comparisons. But we shrink with aversion from such a course. Let it be ours rather to cover with a mantle of charity these shortcomings of our British neighbors, and to admit frankly that we too have sinners on this side of the water. Let it be ours to urge that they take warning from our example, lest a worse state of affairs overcome them. DeQuincey first characterized the man who began by committing murder, and concluded by becoming the worst of confirmed procrastinators. If the British Register be not thoroughly purged, the dreadful consequence may be, that the men who have set an example to others by the commission of the crimes named above, may be followed by others who actually misspell their mother tongue!

